# An Underreported Consequence of Obesity in Pregnancy: Patient-Prosthesis Mismatch

**DOI:** 10.1155/2012/918352

**Published:** 2012-07-08

**Authors:** William R. Hartman, Katherine W. Arendt, Kent H. Rehfeldt

**Affiliations:** ^1^Department of Anesthesiology, Mayo Clinic, Rochester, MN 55905, USA; ^2^Division of Cardiology, Mayo Clinic, Rochester, MN 55905, USA

## Abstract

As the rate of obesity increases in childbearing-aged women, so too will the complications of obesity in pregnancy. An uncommon and likely underreported complication occurs in obese women who have received prepregnancy cardiac valve replacement with a prosthesis that is inadequately sized for body habitus, a condition referred to as patient-prosthesis mismatch (PPM). The physiologic changes of pregnancy as well as the increased weight gain combine to exacerbate PPM. We report a case of PPM that necessitated prosthesis replacement at 16-week gestation. As the incidence of this clinical scenario increases, it is important to understand the implications of prosthesis sizing, as well as the repercussions of having cardiopulmonary surgery to correct the undersized valve prosthesis while pregnant.

## 1. Introduction 

The prevalence of obesity is increasing among women in the United States. In fact, women aged 20–34 have had the largest increase in national obesity rate. As a result, currently 1 in 2 women of childbearing age are overweight (BMI 25.0–29.9) or obese (BMI > 30 kg/m^2^) [1], and approximately 20% of women are considered obese [2] at the beginning of pregnancy. Maternal complications associated with obesity in pregnancy include gestational diabetes, preeclampsia, a higher rate of caesarian delivery, and increased postpartum complications [3].

A likely underreported consequence of obesity in pregnancy is the occurrence of patient-prosthesis mismatch (PPM) [4]. PPM occurs when a cardiac valve prosthesis is of insufficient size for the patient in whom it is inserted (i.e., the prosthesis is too small for the patient) [5]. Most commonly reported following aortic valve replacement (AVR), PPM is associated with inferior hemodynamics, less regression of left ventricular hypertrophy, an increased incidence of cardiac events, and higher mortality rates [6–8]. To date, there is only a single case report describing PPM and pregnancy. Here we present a case in which a morbidly obese woman underwent repeat aortic valve replacement during pregnancy because of worsening heart failure caused by PPM. Although surgery for prosthetic valve dysfunction during pregnancy has been previously described, no case of cardiac surgery during pregnancy to correct PPM has been described.

## 2. Case 

The patient described in the following case has provided written consent for publication of her clinical scenario. A 33-year-old gravida 4, para 0 morbidly obese woman (BMI = 49 kg/m^2^) presented at 16-week gestation with symptoms of worsening heart failure. At the time of presentation, she was complaining of decreased exercise tolerance (it was becoming difficult for her to travel up more than 4 or 5 stairs before stopping to rest), periods of dyspnea at rest, and 2-3 pillow orthopnea. These symptoms were new as she was reportedly relatively asymptomatic from a cardiac standpoint prior to pregnancy.

 Atage 25, she had undergone aortic valve replacement (AVR) with a 19 mm St. Jude mechanical prosthesis for a congenital bicuspid aortic valve. Though her cardiac surgery and subsequent recovery and rehabilitation were successful, the small size of the aortic prosthesis resulted in relative stenosis as her weight progressively increased in the years following surgery. Complicating her recovery, she was diagnosed with systemic lupus erythematous (SLE) and was treated with prednisone, which further contributed to weight gain.

**Figure 1:**
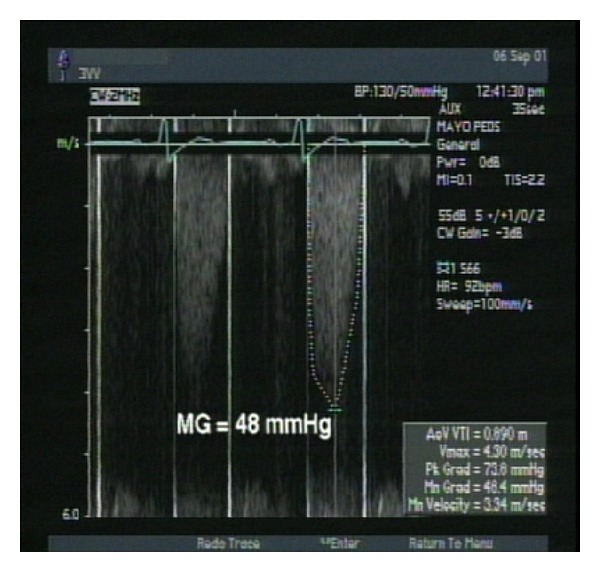
Continuous-wave Doppler recording from the preoperative transthoracic echocardiogram. This Doppler recording from the apical position demonstrates a mean gradient (MG) of 48 mmHg across the aortic valve prosthesis.

This patient had experienced three previous first-trimester miscarriages and at the time of presentation was 16 weeks pregnant. The causes of these miscarriages were undetermined. However, given this history and her worsening symptoms, cardiology evaluation was recommended. A transesophageal echocardiogram (TEE) was performed and demonstrated concentric left ventricular hypertrophy (LVH) with normal systolic function. The aortic prosthesis appeared structurally normal yet the mean transvalvular gradient was elevated at 48 mm Hg with an effective orifice area indexed to body size (EOAI) of 0.26 cm^2^/m^2^, consistent with severe PPM (Figure 1). Upon consultation with the obstetric team, it was recommended that the patient undergo a repeat procedure to replace her undersized aortic prosthesis. Following full discussion of the risks and benefits of repeat cardiac surgery during pregnancy, the patient agreed to proceed. 

**Figure 2:**
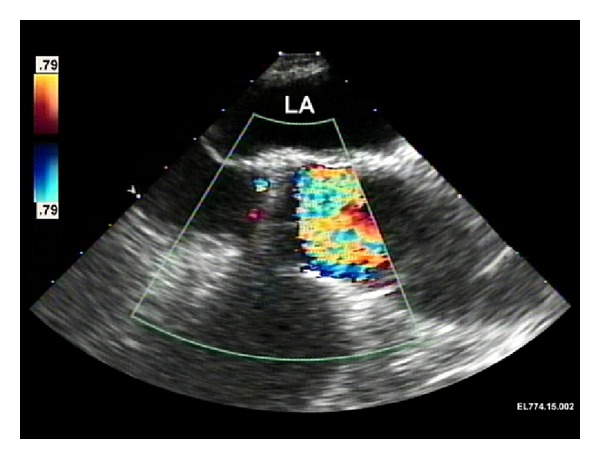
Intraoperative transesophageal echocardiographic view prior to replacement of the aortic valve prosthesis. This midesophageal aortic valvelong axis view during systole demonstrates turbulent colorflow on the aortic side of the St. Jude prosthesis, suggestive of an elevated velocity and prosthetic gradient. The left atrium (LA) is also identified.

**Figure 3:**
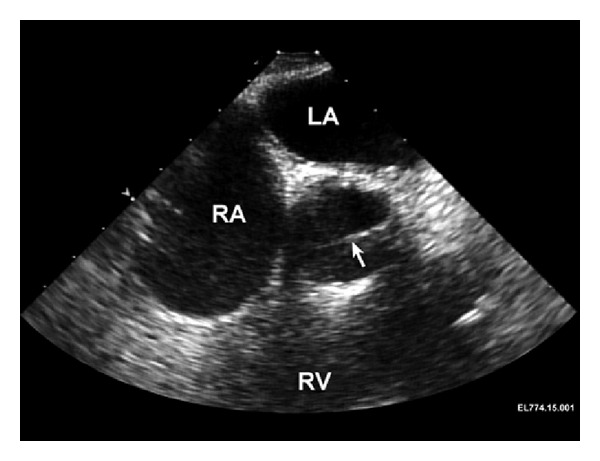
Intraoperative transesophageal echocardiographic view prior to replacement of the aortic valve prosthesis. This midesophageal aortic valve short axis view during systole demonstrates the normal open position of one of the mechanical aortic valve leaflets (arrow). The left atrium (LA), right atrium (RA), and right ventricle (RV) are also identified.

**Figure 4:**
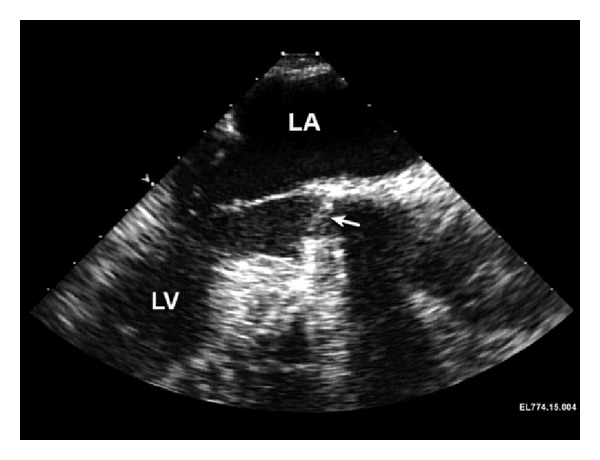
Intraoperative transesophageal echocardiographic view prior to replacement of the aortic valve prosthesis. This midesophageal aortic valve long axis view during systole demonstrates a normal-appearing St. Jude aortic prosthesis during diastole (arrow). In particular, no thrombus, pannus, or mechanical obstruction is identified. The left atrium (LA) and left ventricle (LV) are also identified.

Following ultrasound confirmation of reassuring fetal heart tones, the induction of anesthesia proceeded uneventfully. Standard lines and monitors (arterial line, central venous line, and pulmonary artery catheter) as well as a TEE probe were placed. Following heparinization and insertion of the aortic and venous cannulae in the usual manner, nonpulsatile, normothermic cardiopulmonary bypass (CPB) was initiated. Intraoperative transesophageal echocardiogram images were made prior to AVR replacement demonstrating elevated flow velocity (Figure 2) through an otherwise normal-appearing St. Jude aortic valve prosthesis (Figures 3 and 4). The 19 mm mechanical prosthesis was removed, and the aortic root was enlarged in order to accommodate a 23 mm tissue prosthesis. The patient was weaned from CPB without the need for inotropic support. Anticoagulation was reversed, and surgical closure was performed. A postbypass echocardiogram was performed demonstrating that the aortic valve was well seated with no periprosthetic regurgitation. There was no valvular regurgitation. The mean gradient across the aortic valve was 8-9 mm Hg. These measurements were made at systemic blood pressures of 105/60 mm Hg. The left ventricular systolic function appeared normal. The ascending aorta appeared normal in size without any evidence of dissection or hematoma. The patient was transferred to the intensive care unit where she was weaned from ventilatory support and extubated. Fetal heart rate monitoring was performed throughout the postoperative period and was reassuring. 

The patient underwent an unremarkable recovery and was discharged to home. While the remainder of her obstetric care was provided elsewhere, the patient was reported to have had an otherwise unremarkable term pregnancy, and her infant was born via an uncomplicated caesarian section delivery. The patient has since been lost to followup.

## 3. Discussion

This case highlights a rare but potentially severe consequence of obesity in high-risk obstetric patients with previous cardiac valve replacements. The national rate of obesity in child-bearing age women continues to increase and outpace other demographics. As the obesity crisis continues, so too will the problems associated with obesity in pregnancy. As this case demonstrates, an unforeseen complication of obesity in pregnancy might occur in women with previous cardiac valve replacements. In this case, the pregnancy plus the prosthesis mismatch caused severe heart failure in a pregnant woman, severe enough to require a valve replacement during the pregnancy. Our discussion will highlight points concerning patient prosthesis mismatch and cardiopulmonary bypass surgery during pregnancy.

### 3.1. Patient-Prosthesis Mismatch

In a recent study, severe PPM was shown to occur in 4–10% of casesfollowing aortic valve replacement. An important hemodynamic consequence of PPM is the generation of high transvalvular gradients. In addition to creating an iatrogenic physiologic aortic stenosis, this increased gradient immediately after aortic valve replacement (AVR) surgery delays the regression of left ventricular hypertrophy (LVH). LVH regression is an important predictor of survival after AVR [7]. 

Due to the presence of a sewing ring and artificial leaflet support, all prosthetic heart valves are relatively stenotic when compared with normal valves. However, physicians diagnose PPM when the EOAI falls below 0.85 cm^2^/m^2^ [9]. Moderate PPM is said to exist when the EOAI is between 0.65 and 0.85 cm^2^/m^2^ while an EOAI below 0.65 cm^2^/m^2^ defines severe PPM. In the case we describe, the EOAI was 0.26 cm^2^/m^2^, well within the severe range. The EOA of a prosthetic valve varies with prosthesis size and type. Values of EOA for a variety of prostheses are readily available in the literature [9]. When selecting the type and size of prosthesis, consideration should be given to the EOAI that will result in a given patient. Surgeons typically measure the size of the valve annulus in vivo using calibrated sizing instruments. The annular size is also commonly measured preoperatively with transthoracic echocardiography or TEE. If the presence of a small annulus precludes insertion of an adequately sized prosthesis, consideration can be given to surgical enlargement of the annulus [9].

With the obese population continuing to rise in America, it is likely that more peripartum patients will present with symptoms of PPM. It has been recommended that these patients be followed very closely during their pregnancy with serial echocardiograms. If the heart failure becomes too severe, as in our case, it may become necessary to consider valve replacement surgery to correct PPM. Because the physiologic changes of pregnancy begin in the first trimester and continue to rise into the third trimester, it may not be possible to delay cardiopulmonary bypass surgery until after delivery. In fact, a systematic review from 1984 to 1996 evaluated fetal and maternal outcomes in cardiac surgery performed during pregnancy, immediately after delivery, and after resolution of the postpartum state [10]. Findings suggested that, though potentially beneficial for the fetus, delaying cardiac surgery until after delivery increased maternal mortality. Therefore, performing necessary cardiac surgery while pregnant is in the best interest of the mother with severe cardiac disease. However, these data also indicated that fetal mortality is as high as 30%. The use of appropriate parameters of CPB perfusion may diminish this risk to the developing fetus. We discuss several of these parameters here.

### 3.2. Optimal Gestational Age

This patient underwent CPB at 16-week gestation. No relationship between gestational age at the time of CPB surgery and fetal morbidity and mortality has been conclusively determined. In retrospective series, fetal mortality has been described during every trimester of gestation [10–14]. Nonetheless, many anesthesiologists, cardiologists, obstetricians, and cardiothoracic surgeons recommend that surgery, especially surgery requiring CPB, be delayed until after organogenesis of the fetus during the first trimester of pregnancy. During late second trimester, the cardiac output of the parturient peaks. As a result, the increasing symptoms reported by our patient at the beginning of second trimester would have likely continued to progress. Therefore, early second trimester was the ideal time to perform surgery, preventing further deterioration of cardiac status but exposing the fetus to anesthesia and CPB after organogenesis has occurred. 

### 3.3. Fetal Heart Rate Monitoring Intraoperatively

At early gestational ages, continuous fetal heart rate monitoring can be technically difficult and provide little valuable information. As gestational age nears that of viability, however, some experts recommend fetal heart rate monitoring during CPB [15]. Fetal heart rate (FHR) is related to fetoplacental sufficiency. Acute fetoplacental insufficiency results in fetal bradycardia. Long-term insufficiency results in subsequent fetal acidosis which results in fetal tachycardia with minimal beat-to-beat variability on FHR tracing. 

The initiation of CPB is typically characterized by fetal bradycardia in response to an increase in perfusion pressure. At the conclusion of CPB fetal tachycardia with reduced beat-to-beat variability may be observed [16, 17]. Further, hypothermia causes fetal bradycardia and if the patient is cool, the patient could be warmed. Some argue that FHR monitoring is not helpful during CPB in that pregnant patients under CPB should simply be maintained normothermic and on maximal perfusion pressure throughout the CPB run. Therefore, if fetal bradycardia occurs during surgery, there is no further intervention possible. Cesarean delivery during CPB is not recommended because of anticoagulation and the risk of exsanguination to the mother.

### 3.4. Cardiopulmonary Bypass Technique: Normothermic versus Hypothermic

Hypothermia should be avoided during CPB in the pregnant patient because it likely causes decreases in placental blood flow and oxygen transfer, and it may cause increases in uterine contractions and episodes of fetal bradycardia and asystole. Most importantly, though, it has retrospectively been shown to increase fetal mortality. In a review of 69 cases from 1958 to 1992, the authors found that in the 40 most recent cases of pregnant women undergoing CPB, fetal mortality was 24% in the hypothermic CPB group and zero in the normothermic CPB group [18]. Therefore, maintenance of normothermia is important for fetal well-being.

## 4. Conclusion

PPM occurs when a valvular prosthesis is insufficient in size for the intended patient. In this case, a 19 mm aortic prosthesis was placed in a woman who subsequently became morbidly obese and later, pregnant. The hemodynamic changes of pregnancy,specifically the increased cardiac output,contributed to worsening heart failure, and she required cardiopulmonary bypass surgery early in her second trimester. Recommendations for successful cardiac surgery in the pregnant patient include optimizing the gestational age at the time of surgery, the prudent use of fetal heart rate monitoring, high flow, and normothermic CPB [13]. In our case, replacement of the valve resulted in a favorable outcome for both mother and fetus.
